# PP71 Organizations Responsible For The Evaluation Of Health Technologies Globally: A Scoping Review

**DOI:** 10.1017/S0266462324002411

**Published:** 2025-01-07

**Authors:** Celina Borges MigliavacaCelina Borges Migliavaca, Verônica Colpani, Miriam Allein Zago Marcolino, Maicon Falavigna, Carisi Anne Polanczyk

## Abstract

**Introduction:**

The process of health technology assessment (HTA) is a valuable tool for the pursuit of equitable and sustainable healthcare systems. Various countries have established organizations dedicated to conducting HTAs, adapting such institutions to local healthcare ecosystems. The aim of this study was to evaluate the structure, methods, and processes of organizations responsible for national-level HTAs globally.

**Methods:**

A scoping review was conducted assessing organizations responsible for conducting HTAs for national-level decision-making in any country. Identification of eligible organizations was performed through a review of member organizations of INAHTA, EUnetHTA, RedETSA, and HTAsiaLink networks, as well as organizations evaluated in reviews with a similar scope. For each organization, the following data were searched: country, year of foundation, organizational nature, role in decision-making, funding, technologies assessed, criteria considered for decision-making (such as efficacy and safety, costs, impact on equity, among others), type of economic evaluation, and patient involvement.

**Results:**

We identified 69 organizations, from 56 countries, mainly European (n=39; 56%). Fifty-three (77%) are government-affiliated; most (n=51; 74%) have a consultative role. Public funding is the main funding, and 12 (17%) organizations charge fees for conducting HTA. Technologies assessed include drugs (n=61; 88%), devices (n=47; 68%), and procedures (n=33; 48%). HTA is usually initiated upon request from the manufacturer (n=45; 65%). Patient involvement is not clearly described in 32 organizations (46%); in 29 organizations (42%), the role of patients is to provide information that is considered during decision-making.

**Conclusions:**

Among the evaluated organizations, it is observed that the majority are government-affiliated, have public funding, and play a consultative role. The results of this study serve as an important reference for the development and improvement of organizations responsible for conducting HTAs.

